# Where, When and What? A Time Study of Surgeons' Work in Urology

**DOI:** 10.1371/journal.pone.0092979

**Published:** 2014-03-24

**Authors:** Jan Wolff, Paul McCrone, Gerd Auber, Petra Fiedler, Anita Patel, Ulrich Wetterauer

**Affiliations:** 1 University Medical Centre Freiburg, Department of Management and Controlling, Freiburg, Germany; 2 King's College London, Institute of Psychiatry, Health Service and Population Research, Centre for the Economics of Mental and Physical Health, London, United Kingdom; 3 University Medical Centre Freiburg, Department of Urology, Freiburg, Germany; School of Population Health, The University of Queensland, Australia

## Abstract

**Introduction:**

Staff time is a relevant resource in the delivery of health care interventions. Its measurement is a prerequisite for unit costing but usually complex. The aim of this study was to analyse the distribution of surgeons' work time among types and places of activities. A second aim was to use these data to calculate costs per unit of output.

**Methods:**

A self-reporting work sampling study was carried out at a department of Urology. All of twelve surgeons involved in clinical care participated in a two-week analysis of their work time.

**Results:**

A total of 2,485 data-points were collected, representing about 1,242 hours of work time. Surgeons spent the greater part of their work time in direct patient care, but substantial shares were required for documentation and organisation. Assistants were mainly required at the wards and consultants at the operating theatre and the outpatient unit. Staff costs of surgeons were 32 € and 29 € per patient day at the wards, respectively, 1.30 € per minute at the operating theatre and 32 € per visit at the outpatient unit.

**Conclusion:**

Results provided a basis for costing of health care interventions at the study site. However, future research should focus on the establishment of standardised terminology in order to increase transferability of results.

## Introduction

Costing of health care interventions requires an adequate measurement of relevant resource use [1]. Staff costs account for around two-thirds of overall spending on health care in the National Health Service (UK) [2]. However, measurement of staff time use appears to be inherently more complex than measurement of material resources, since movements of men “leave nothing visible or tangible behind them” (Taylor, 1919) [3]. In particular, an estimate of time allocation is a prerequisite for costing of output if staff is not solely located at one functional unit of care but changes frequently. This is the case in disciplines at the intersection of surgery and internal medicine, such as Urology.

The relevance of studies aiming to quantify staff time decreased in industrial economics since automation has mainly reduced dispersion in resource use [4]. In contrast, health care processes are less standardised than industrial processes, due to factors such as professional discretion, complexity and unpredictable needs of patients [5].

Two main approaches in measuring staff time in health care are continuous observations and work sampling, i.e. snapshots of activities at random instants [6]. Tipping et al (2010) [7] have systematically reviewed the literature reporting such studies of physicians' time in an inpatient setting. They have found eleven time-and-motion studies and two work-sampling studies. However, identified studies were mainly focused on physicians in training and of varying quality.

The main aim of this study was to analyse the distribution of licensed surgeons' work time among types and places of activities and its differences between assistant and consultant surgeons and between different times of the day. A second aim was to use these data to calculate costs of surgeons per unit of output.

## Methods

A self-reporting work sampling study was carried out at the department of Urology in the surgical hospital at the Academic Medical Centre Freiburg. The department has two wards with 22 beds each. It provides care to about 2,500 inpatient and 10,700 outpatient episodes and conducts about 7,000 surgical interventions per year.

All of twelve surgeons involved in clinical care at the department of Urology participated in a two-week analysis of their work time, equally divided into six assistant and six consultant surgeons. A mobile device carried by each participant gave random requests for documentation within 30-minute intervals. Upon request, participants documented the current type and place of activity they were carrying out. Measurement included the whole workday but excluded on-call duties. The study was carried out during semester break, meaning there were less academic activities than usual. Participation was voluntary. Consent was given in advance and confirmed with handing in the documentations. There were no non-responders. The study was completely anonymous and its background and intention were clearly described beforehand to reduce observer effects. The study and its methodology were approved by the works council of the University Medical Centre Freiburg and by the ethics committee of the Albert-Ludwigs-University of Freiburg.

The number of surveyed categories was kept small in order to reduce complexity and burden on participants, while keeping it sufficiently large to calculate unit costs. Surveyed places comprised two wards, an operating theatre, an outpatient unit and a category summarising all other places. The activity type patient care comprised all actions in direct contact with the patient, such as medical examination and surgical operations. Documentation and organisation comprised activities required to supplement and maintain clinical activities without contact to the patient, such as writing medical reports and planning of interventions. Research comprised all activities that were carried out in the context of scientific investigations, such as laboratory work or writing of papers. In cases of overlapping actions, such as clinical research in contact with the patient, the participants were informed to classify according to the main purpose of the task. The category ‘others’ comprised all other non-clinical activities, such as teaching, transit times and breaks.

Statistical analysis included maximum-likelihood estimations for both total proportions of work time and differences in proportions between subgroups. Blyth-Still-Casella [8], [9] confidence intervals were calculated for total proportions and Agresti-Min [10] confidence intervals were calculated for differences between subgroups.

In order to calculate costs of surgeons per unit of output, work time distributions were weighted by average wage differences between assistant and consultant surgeons according to collective bargaining agreements. Total costs of surgeons were allocated to functional places, i.e. wards, operating theatre and outpatient unit, according to the weighted work time distribution. Time shares in the category ‘others’ were allocated to functional places in relation to primary work time distribution, meaning a place with 30% of primary work time would receive twice as much allocation as one with 15% primary work time. Allocated costs were divided by total output in order to calculate unit costs. Number of patient days at the wards and visits at the outpatient unit were derived from the patient administration database. Total minutes at the operating theatre were derived from the electronic hospital information system, where duration between first cut and final suture and number of participating surgeons were documented for each intervention.

## Results

A total of 2,485 documentations of type and place of activities were made, representing about 1,242 hours of work time. Table 1 shows the distribution of total work time among types and places of activities.

**Table 1:** Work time distribution of surgeons among types and places of activities.

	ward 1	ward 2	operating theatre	outpatient unit	other	total
patient care	**7.08%**	**7.04%**	**23.58%**	**22.78%**	**3.98%**	**64.47%**
	(6.11% to 8.14%)	(6.07% to 8.11%)	(21.92% to 25.27%)	(21.14% to 24.48%)	(3.25% to 4.8%)	(62.57% to 66.35%)
documentation/organisation	**5.03%**	**3.46%**	**0.28%**	**8.97%**	**7.53%**	**25.27%**
	(4.2% to 5.96%)	(2.78% to 4.24%)	(0.13% to 0.56%)	(7.88% to 10.17%)	(6.52% to 8.6%)	(23.59% to 27.00%)
research	**0.00%**	**0.00%**	**0.00%**	**0.12%**	**5.43%**	**5.55%**
	(0% to 0.14%)	(0% to 0.14%)	(0% to 0.14%)	(0.03% to 0.34%)	(4.58% to 6.37%)	(4.69% to 6.52%)
other	**0.00%**	**0.00%**	**0.00%**	**0.32%**	**4.39%**	**4.71%**
	(0% to 0.14%)	(0% to 0.14%)	(0% to 0.14%)	(0.14% to 0.62%)	(3.62% to 5.24%)	(3.94% to 5.59%)
total	**12.11%**	**10.50%**	**23.86%**	**32.19%**	**21.33%**	
	(10.85% to 13.43%)	(9.33% to 11.75%)	(22.2% to 25.58%)	(30.36% to 34.05%)	(19.73% to 22.98%)	

percentages: maximum-likelihood estimator; parentheses: Blyth-Still-Casella intervals.

Almost two-thirds of total work time was devoted to direct patient care and more than one-fourth was required for documentation and organisation. Six per cent of work time was devoted to research. The remainder was spent for other activities, such as teaching, time to move from one working place to the other and breaks. Surgeons equally divided half of their working time in activities at the wards (22.6%) and activities at the operating theatre (23.9%). Almost one third of total work time was spent in the outpatient unit. The remainder was spent in other places, such as offices or meeting rooms.

There were substantial differences in time shares required for different tasks between places of activities. Thirty-eight per cent of total work time spent at the wards was required for tasks in the group of documentation and organisation. In contrast, time spent at the operating theatres was almost entirely designated to direct patient care.

Table 2 shows the distribution of work time among places stratified by professional groups, i.e. assistant and consultant surgeons. Assistants spent the main part of their work time (41%) at the two wards. Consultant surgeons spent significantly larger shares in operating theatres, the outpatient unit and other places. There were no significant differences in shares of time devoted to activity types, except of time in research, where assistants spent higher percentages (not shown in table).

**Table 2:** Difference in work time allocation between assistant and consultant surgeons

	consultant	assistant	absolute difference
ward 1	3.58%	21.67%	−18.09%
			(−20.74% to −15.57%)
ward 2	2.74%	19.20%	−16.46%
			(−18.96% to −14.08%)
operating theatre	27.95%	19.28%	8.67%
			(5.33% to 11.98%)
outpatient unit	36.25%	27.65%	8.61%
			(4.94% to 12.25%)
other	29.47%	12.20%	17.27%
			(14.16% to 20.38%)

percentages: maximum-likelihood estimator; parentheses: Agresti-Min intervals.

**Figure 1:**
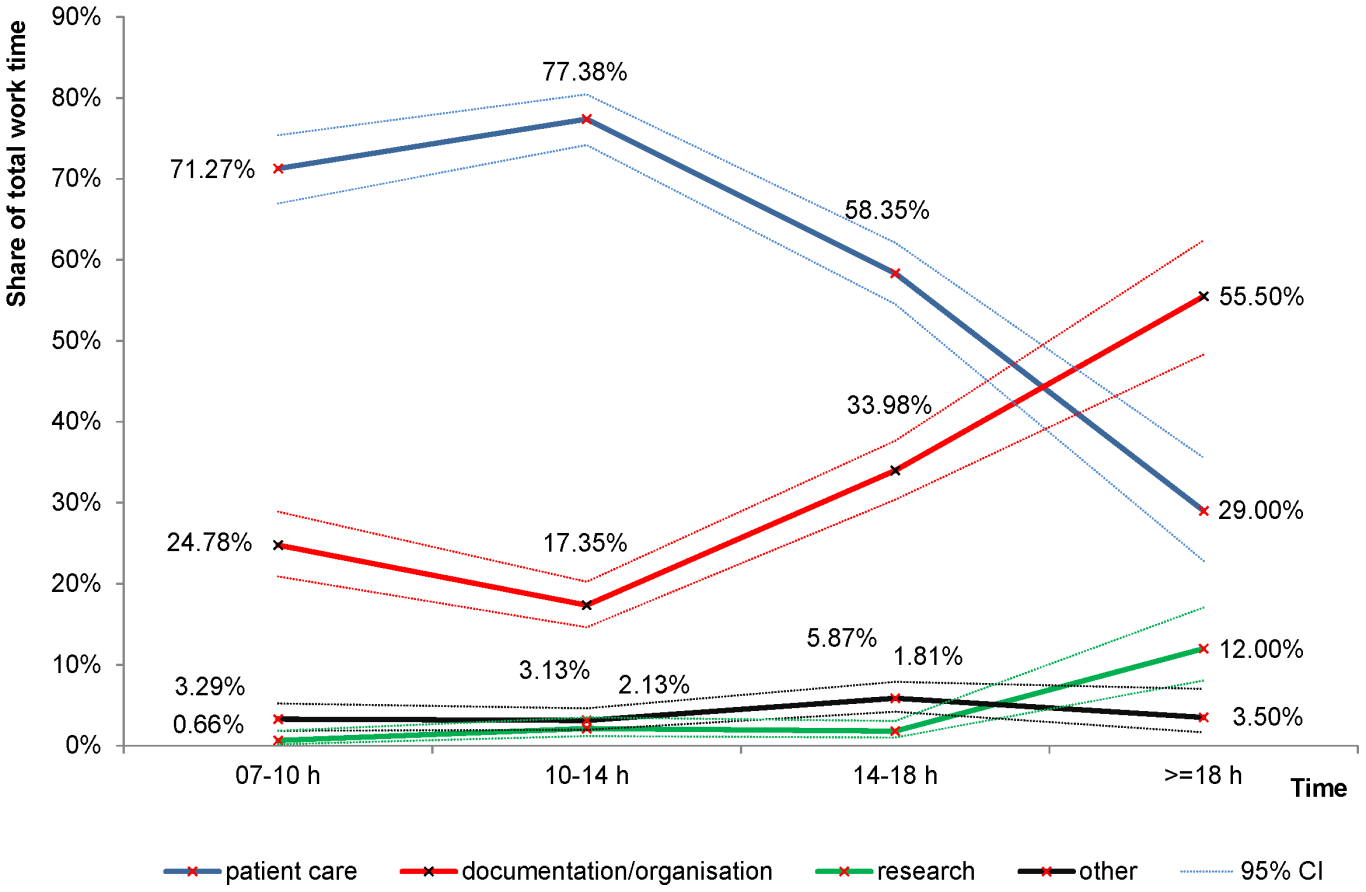
Work time distribution among activity types and times of the day.

Figure 1 illustrates the distribution of work time among activity types during the day. While direct patient care accounted for almost 80% of working time between 10am and 2pm, it declined to less than 30% after 6 pm. On the other hand, documentation and organisation required less than 20% of total working time between 10am and 2 pm and climbed up to 56% after 6 pm. Research was almost entirely carried out after 6pm.

**Figure 2:**
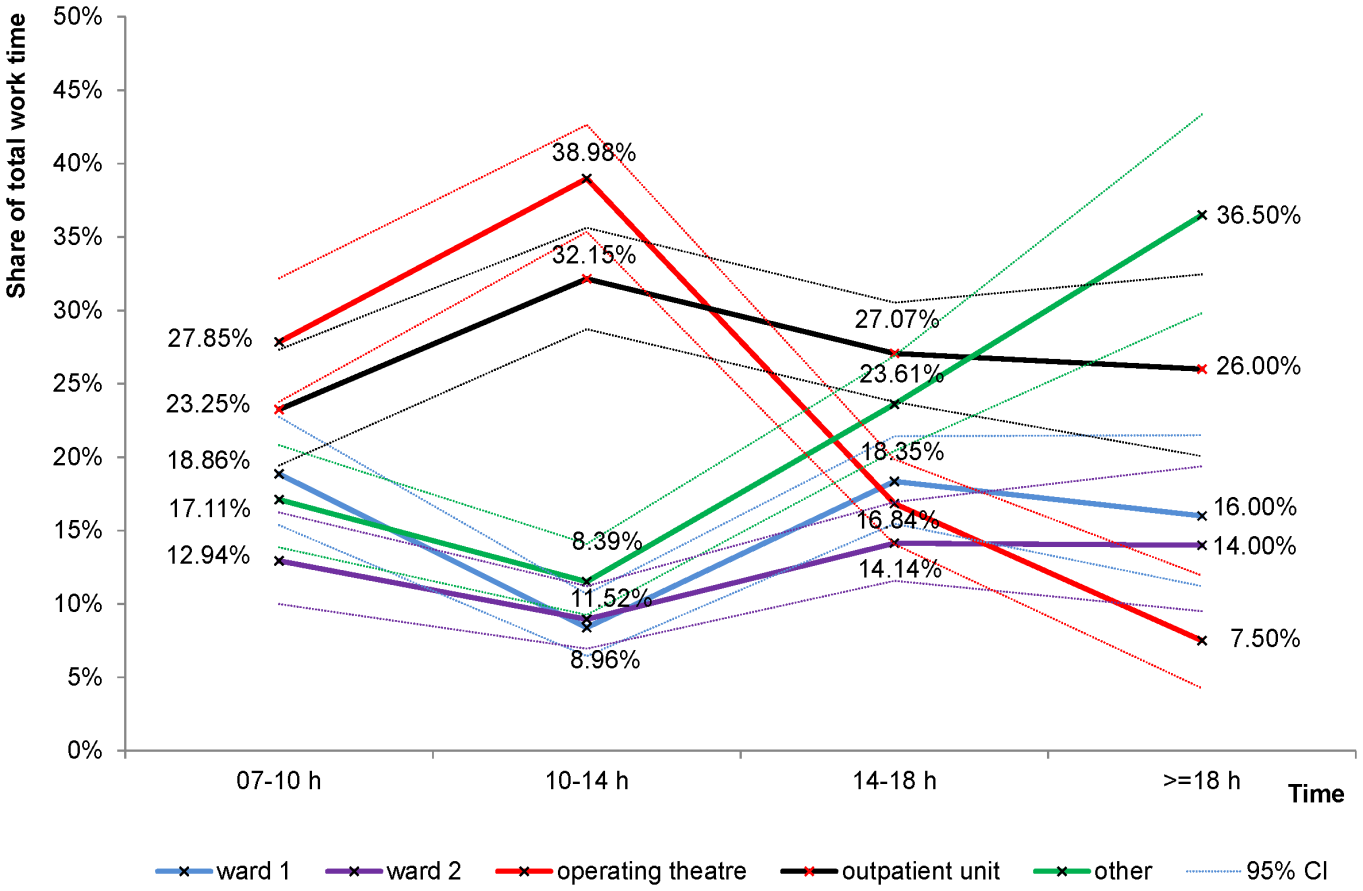
Work time distribution among activity places and times of the day.

Figure 2 illustrates the distribution of work time among places during the day. Activities at the two wards in sum required the largest share between 7am and 10am (32%) and declined to 20% between 10am and 2pm. The share of time spent in the operating theatre increased from 28% between 7am and 10am to 39% between 10am and 2pm.

Table 3 shows costs of surgeons per unit of output at different places. Despite similar costs per day for patient care, total costs per day at ward 1 were 13% higher than total costs per day at ward 2, as a result of higher costs for documentation and organisation.

**Table 3:** Costs of surgeons per unit of output.

	patient care	documentation/organisation	total
ward 1 (per patient day)	19.83 €	12.39 €	32.22 €
ward 2 (per patient day)	19.91 €	8.60 €	28.50 €
operating theatre (per minute)	1.28 €	0.02 €	1.30 €
outpatient unit (per visit)	22.46 €	9.10 €	31.56 €

## Discussion

The main aim of this study was to analyse the distributions of surgeons' work time among types and places of activities and to analyse whether these distributions differ between assistant and consultant surgeons and between different times of the day. A second aim was to use these results to calculate staff costs per unit of output. Surgeons spent the greater part of their work time for direct patient care, but substantial shares were required for documentation and organisation. These results were common to assistant and consultant surgeons. Significant differences between the two groups were found in time shares spent at different locations, with assistants being mainly required at the wards and consultants at the operating theatre and the outpatient unit.

The results allowed calculating staff costs per output at different functional places. This revealed aspects relevant to decision makers for potential reorganisation, such as higher costs related to documentation and organisation at ward 1 than at ward 2. Despite slightly overlapping confidence intervals, the difference in work time in this category between the two wards was statistically significant. A possible explanation for higher percentages in the group of documentation and organisation is the shorter average length of stay of patients admitted to ward 1 than of patients admitted to ward 2 (ward 1: mean 4.8 days, median: 3 days; ward 2: mean 5.3 days; median: 3 days). However, despite statistical significance, the clinical relevance of the difference in length of stay was not very high. Another possible explanation might be mere differences in the structuring of administrative processes between the two wards, meaning higher efficiency at ward 2.

The percentage of total time spent in direct care (64%) appears high in comparison to previously conducted studies. Most studies in Tipping et al [7] found smaller percentages, ranging from 41% to 8% of total staff time. However, included studies were focused on non-licensed physicians and activities in the context of receiving education accounted for up to 21% of total work time.

Moreover, the share of direct patient care appears to be higher in surgery than in other medical disciplines. Mache et al carried out two time studies with similar methods in surgical [11] and in gastroenterological wards [12], respectively. They found junior surgeons' share in direct patient care (44%) to be more than twice the share of internists. This finding is also epistemologically coherent with very high shares of direct patient care that were found in this study during presence in the operating theatre (Table 1). Weigl et al (2010) [13] found similar results, with activities in the operating theatre accounting for almost a four-fold share in direct patient care (75%) in comparison to activities at the ward.

Dissimilarities in results might have been true reflections of differences in clinical practices and different semantics in study design. In addition, there are three main caveats in conducting work sampling studies, which could have influenced internal validity [14].

First, the study period might not have been representative for the usual activities at the department and statistical significance might not represent true uncertainty, since real variations from one time to the other exist. In particular, the study period was during the semester break, meaning shares of time in teaching activities but also in research were less than should be expected in regular periods. Beyond this aspect, the study period should be mainly representative of usual circumstances, since measurements were extended over an adequate length of time, i.e. 14 days, which should be sufficient according to common standards [15]. Moreover, staff reported that the study period was not different to usual circumstances beyond absence of students.

Second, ‘snap-shots’ of staff activities were taken instead of continuous observations and these are vulnerable to random errors of observations. However, the sample size was large enough to mainly alleviate this caveat. Furthermore, calculated Blyth-Still-Casella intervals guaranteed nominal coverage, in contrast to widely used Wald intervals, and were more efficient than others, such as Clopper-Pearson intervals [16].

Third, participants might have changed their behaviour, maybe subconsciously, as a mere result of their knowledge about being investigated. This potential caveat was addressed by strict anonymisation of data, clear presentation of aims and objectives of the inquiry in advance and early involvement of staff representatives in conceptualisation of the study. Nevertheless, it is impossible to completely rule out the possibility of bias induced by such effects.

This study provided estimates of staff time distributions among types and places of activities. These allowed costing of output delivered at the department of Urology, which was impossible before accurate allocation of staff costs. Future research should focus on the development of standardised definitions of staff activities in order to increase generalisability and transferability of results.
